# Penile Mondor’s in a Covid-19 patient on prophylactic anti-thrombosis with rivaroxaban: a case report

**DOI:** 10.1186/s12301-021-00200-7

**Published:** 2021-07-06

**Authors:** Murat Tuğrul Eren, Hakan Özveri, Hilal Kurtoğlu

**Affiliations:** 1 Department of Urology, Acıbadem Healthcare Group, Acibadem Kozyatağı Hospital, 19 Mayıs, Kozyatağı Kavşağı No:24, 34734 Kadıköy/İstanbul, Turkey; 2 Vocational School of Health Sciences, Head of Surgical Technician Programme, Acıbadem Mehmet Ali Aydinlar University, Istanbul, Turkey; 3 Department of Urology, School of Medicine, Acıbadem Mehmet Ali Aydinlar University, Ondokuz Mayıs Mah. Begonya Sk. No: 12, Kadıköy/İstanbul, Turkey; 4 Department of Cardiology, Acıbadem Healthcare Group, Acibadem Hospital, Tekin Sk. No:8, 34718 Kadıköy/İstanbul, Turkey

**Keywords:** COVID-19, Drugs and medicines, Venous thromboembolism

## Abstract

**Background:**

Penile Mondor’s disease (PMD) is thrombophlebitis of the superficial dorsal vein of the penis. Following the occurrence of thrombotic events in the affected veins, the lumen often becomes occluded with fibrin and inflammatory cells. A hyper-coagulative state is one of the underlying causes although most cases of PMD are idiopathic. Coronavirus disease-2019 infection (COVID-19) is associated with frequent thrombotic events. Inflammation and thrombosis play a central role in the course and outcome of COVID-19, which can predispose to both venous and arterial thromboembolism. In this report, we present a 33-year-old male patient diagnosed with PMD during the subacute phase of COVID-19 infection while on prophylactic antithrombotic treatment.

**Case Presentation:**

A 33-year-old male patient was diagnosed as PMD which occurred during the subacute phase of COVID-19 infection, while he was on active treatment of COVID-19 by prophylactic antithrombotic Rivaroxaban 15 mg therapy and curative antiviral medication. There was no recent sexual intercourse or trauma to the genitals. His PCR test for COVID-19 had become negative, and antibody test was positive at the time of his PMD’s onset. Rivaroxaban was replaced by Enoxaparin (8000 IU/0.8 ml.), a low molecular weight heparin administered subcutaneously and twice daily. On the third day of this medication, all coagulative measurements returned to normal. PMD disappeared in the second week.

**Conclusion:**

Low-dose Rivaroxaban 15 mg is not safe for some COVID-19-associated thromboembolism prophylaxis, and careful follow-up is critical due to the possibility of a wide range of pathologic thrombotic manifestations in COVID-19 infection.

## Background

Mondor’s disease is thrombophlebitis of the superficial veins named after the French surgeon Henri Mondor, originally described in the chest wall vessels in the late 1930s [1]. Penile Mondor’s disease (PMD) is thrombophlebitis of the superficial dorsal vein of the penis is usually considered thrombophlebitis or phlebitis of subcutaneous vessels [2].

After the occurrence of thrombotic events in the affected veins, there is often a blockage of the lumen with fibrin and inflammatory cells. A hyper-coagulative state is one of the underlying causes, although most cases of PMD are idiopathic [3, 4].

Coronavirus disease-2019 infection (COVID-19) is associated with frequent thrombotic events. Inflammation and thrombosis play a central role in the course and outcome of COVID-19, which can predispose to both venous and arterial thromboembolism due to excessive vascular and systemic inflammation, endothelial dysfunction, cytokine storm, hypoxia and immobilization [5–11].

The International Society on Thrombosis and Haemostasis (ISTH) recommended a prophylactic dose of low molecular weight heparin (LMWH) in all hospitalized patients with COVID-19 unless contraindicated [12]. However, recent suggestions recommend that new-generation anticoagulants should be preferred because they have a better safety profile, fixed dosing and they do not require laboratory monitoring, which helps limit patient contact with health-care providers, all features that are particularly important for outpatient management in this pandemic [13].

In this report, we present a 33-year-old male patient diagnosed with PMD during the subacute phase of COVID-19 infection while on active treatment of COVID-19 with prophylactic antithrombotic Rivaroxaban 15 mg therapy and antiviral medications after a written consent for publication was obtained from the patient.

## Case Presentation

A 33-year-old married male patient with a body mass index of 28.6 noticed a palpable subcutaneous cord-like induration under the skin of the penis with a mild pain to the touch. When these complaints began, he was on day 14 of medical treatment for COVID-19 infection diagnosed by both “Polymerase Chain Reaction (PCR)” positivity and “low-Dose Computerized Chest Computerized Tomography” (ground glass appearance bilaterally) due to symptoms of high fever, cough, loss of smell and taste without any shortness of breath. His blood count was normal at the diagnosis time of COVID-19 with all parameters including neutrophil/lymphocyte ratio. D-dimer and Ferritin levels were also normal, while alanine transaminase and C-reactive protein levels were slightly elevated (66 IUL/L (Normal: 12–63) and 1.49 mg/dl. (Normal: < 0.5), respectively). His father and two uncles had undergone cardiac stent placement due to vascular pathology, but the patient had no known cardiovascular pathology.

According to the National Ministry of Health algorithms for COVID-19 treatment and after clinical assessment on family history, he was admitted to isolated home treatment with Favipiravir 2 × 1600 mg. (po) followed by a maintenance dose of 2 × 600 mg. for five days along with the prophylactic antithrombotic agent Rivaroxaban 15 mg. (po) once daily. On the 14th day of follow-up, the patient was scheduled for PCR and antibody test for COVID-19, and he was also admitted to urology clinics for his complaint described above. PMD is the initial clinical diagnosis.

**Fig. 1:**
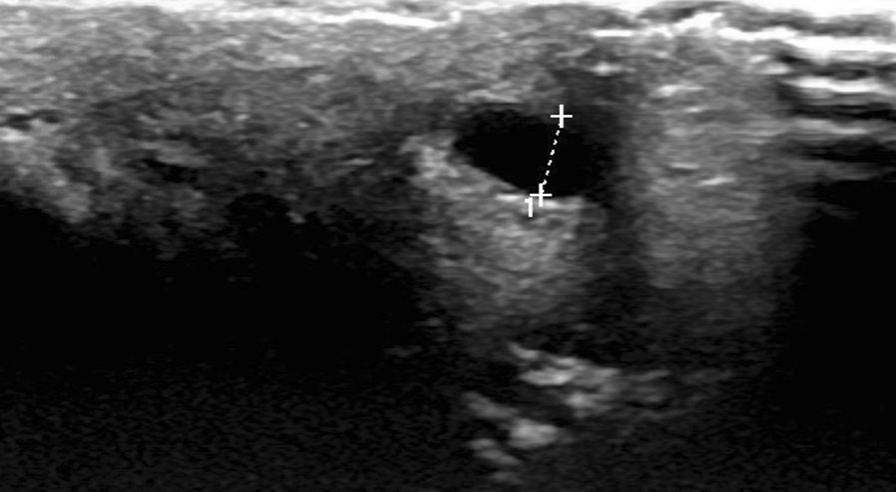
Ultrasonographic view of incompressible dilated superficial venous structure on dorsum of penis

**Fig. 2:**
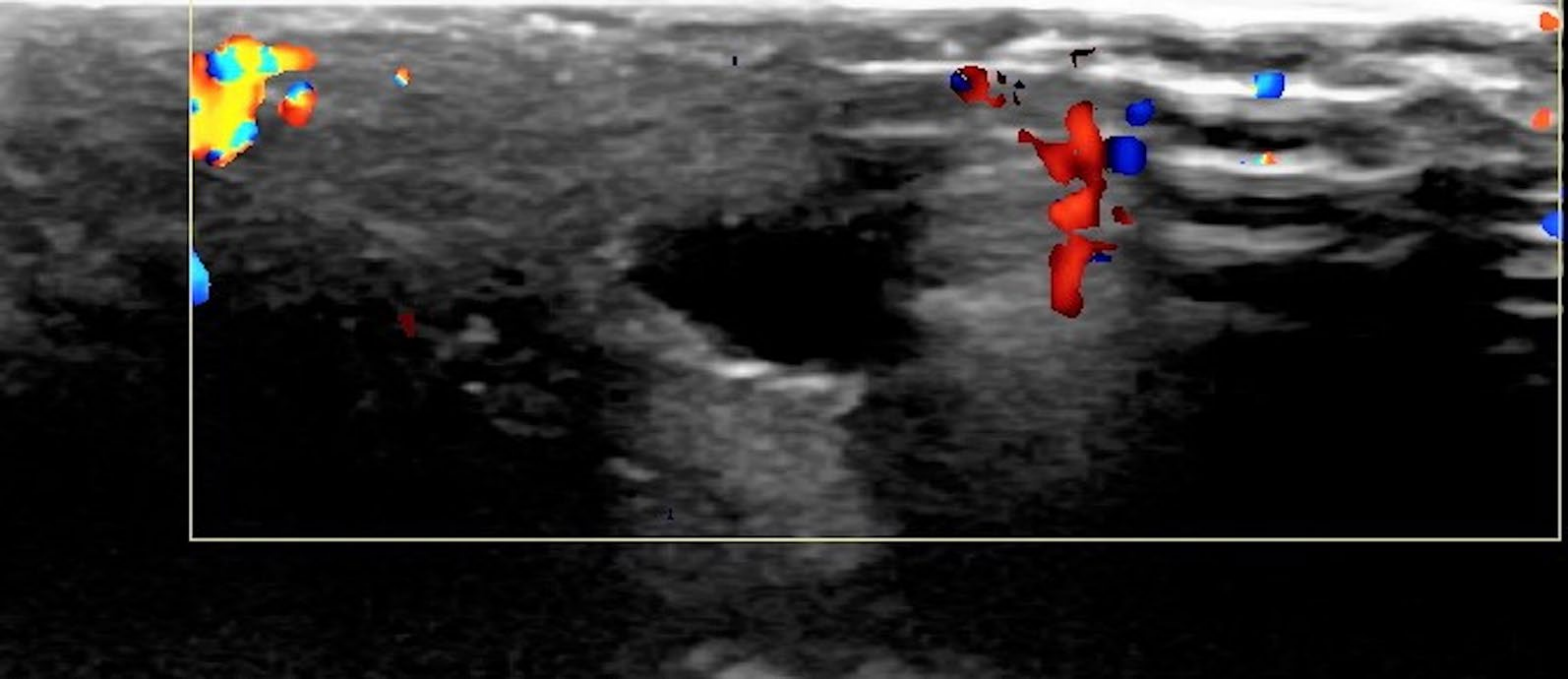
There was no vascularization at the Doppler ultrasonographic evaluation of the same structure indicating an obstructing thrombus

Superficial Doppler ultrasonography revealed dorsal induration consistent with segmental thrombosis of the superficial dorsal vein, suggestive of penile Mondor’s disease (Figs. 1, 2).

The patient was immediately evaluated for thrombophilia for which tests complete blood count, prothrombin time (PT), activated partial thromboplastin time (aPTT), thrombodynamics test, thrombin time (TT) and reptilase time were done after consultation of haematology and cardiology. Lupus anticoagulant, anticardiolipin antibody, anti-β2 glycoprotein 1 antibody, activated protein C resistance, fibrinogen tests, factor V Leiden and prothrombin mutation, MTHFR A1298C mutation, ACE I/D variation and basal homocysteine levels were also evaluated.

The results showed that aPTT was slightly prolonged (36.6 min.-normal range: 21–36 min.) along with PT (15.1 s.-normal range:9.3–14.0 s.). The international normalized ratio (INR) was also increased to 1.3 (Normal = 0.8–1.25), while aPTT was decreased to %60.4 (Normal = 70–130). Genotyping revealed heterozygous variants of MTHFR A1298C and genotype ACE D/D. The other genetic tests were within normal limits.

The PCR test for COVID-19 was negative, and the total antibody for COVID-19 was detectable in the blood test. Low-dose computed tomography of the chest had normalized along with symptoms on the day of admission to urology.

Because of the risk of generalized thrombus associated with Covid-19, Rivaroxaban was replaced with an LMWH, enoxaparin 8000 IU/0.8 ml. administered subcutaneously and twice daily, which is also an appropriate treatment for PMD. On the third day of this medication, all of the patient’s coagulation measurements returned to normal (PT, activated PT and INR). PMD disappeared in the second week of Enoxaparin treatment.

## Conclusions

Following the first report of COVID-19 infection in late December 2019, the scientific community has focused its attention on the impact of this virulent virus on all organ systems [14, 15]. The cardiovascular system is one of the major organ systems of which COVID-19 has a number of important implications [9].

COVID-19 infection can cause susceptibility to thrombotic disease through a mechanism that is not yet fully understood, in both venous and arterial circulation due to blamed causative factors such as excessive inflammation, platelet activation, endothelial dysfunction, and stasis [11]. The manifestations of COVID-19 thromboembolism vary from patient to patient, and excessive thrombosis and embolism can lead to death.

However, thrombotic complications are usually underestimated by clinicians unless they occur in hospitalized patients with severe symptoms associated with infection. Moreover, in patients who do not require hospitalization and are treated at home, disappearance of symptoms with infection and normalization of blood tests including negativity of PCR test with or without formation of antibodies are acceptable for termination of treatment. On the other hand, the severity of the disease is variably associated with prolongation of PT and INR, and TT. There is a trend towards a shortened aPTT, which was present in our patient at the time of PMD diagnosis and PCR negativity. Death from COVID-19 infection is also associated with PT prolongation (by 14%, P < 0.001) and 71% of COVID-19 patients who died met the ISTH criteria for “Disseminated Intravascular Coagulation”, compared with only 0.6% of survivors [11].

We believe that our patient’s PMD was due to COVID-19 infection-related coagulopathy confirmed by blood coagulation tests as there were no other causes of PMD, such as sexual or masturbation trauma, cancer or chronic diseases (vasculitis, etc.). Interestingly, this occurred during treatment with prophylactic antithrombotic medication after the patient recovered from COVID-19 infection-related symptoms along with recovery from laboratory abnormalities. In addition, genetic testing and thrombophilia evaluations found no homozygous pathology. At the time PMD occurred, PCR test for COVID-19 changed from positive to negative and antibodies for it developed at the same time. This may show that systemic involvement of coronavirus infection can continue to progress even though symptoms and laboratory abnormalities normalize.

Studies have shown that hospitalized patients with acute medical conditions are at increased risk for venous thromboembolism (VTE), which can be reduced by prophylactic anti-coagulation [11]. However, the role of administering prophylaxis in outpatients with mild COVID-19 symptoms is uncertain and not routinely used.

Low-dose Rivaroxaban is a new-generation anticoagulant and a factorXa inhibitor that was used for thrombosis prophylaxis in this patient. This could have potentially saved the patient from more severe thrombotic complications. However, we believe that the omission of full-dose Rivaroxaban treatment in this patient despite normal renal function may be the reason for inadequate VTE prophylaxis.

To the best of our knowledge, there is currently only one case report in the literature of a similar presentation in a patient admitted for PMD symptoms and diagnosed as COVID-19 infection along with PMD [16]. This case might differ from our case in two aspects: First, the former patient had PMD at the time of PCR + COVID-19 diagnosis, whereas the latter one had already been diagnosed with COVID-19 viral infection. Second, in our case, PMD manifested at the end of treatment for COVID-19, supported by PCR negativity and antibody formation, and the patient was also taking prophylactic drugs for thrombosis. His symptoms of COVID-19 infection had disappeared when PMD occurred.

In conclusion, low-dose Rivaroxaban 15 mg is not safe for some COVID-19-associated thromboembolism prophylaxis. Because of the wide spectrum of pathologic thrombotic manifestations, careful follow-up is as important as clinical assessment of thromboprophylaxis in selected patients with COVID-19.

## Data Availability

The datasets used and/or analysed during the current study are available from the corresponding author on reasonable request.

## Abbreviations

PMD: Penile Mondor’s disease
COVID-19: Coronavirus disease-2019
ISTH: The international society on thrombosis and haemostasis
LMWH: Low molecular weight heparin
PCR: Polymerase chain reaction
PT: Prothrombin time
aPTT: Activated partial thromboplastin time
TT: Thrombin time
INR: The international normalized ratio
VTE: Venous Thromboembolism
